# Precision leak detection in the golden window: emerging biomarker strategies for anastomotic integrity monitoring after low anterior resection

**DOI:** 10.1097/JS9.0000000000002891

**Published:** 2025-06-25

**Authors:** Zihan Xiong, Jie Liu, Lin Chen, Yang Yang, Song Zhao

**Affiliations:** a Department of General Surgery, The Thirteenth People’s Hospital of Chongqing (Chongqing Geriatrics Hospital), Chongqing, China; b Department of Clinic Laboratory, The Thirteenth People’s Hospital of Chongqing (Chongqing Geriatrics Hospital), Chongqing, China; c Department of Clinical Nutrition, The Thirteenth People’s Hospital of Chongqing (Chongqing Geriatrics Hospital), Chongqing, China

**Keywords:** anastomotic leakage, biomarkers, drainage fluid, early diagnosis, low anterior resection, surgery

## Abstract

Anastomotic leakage (AL) following low anterior resection for rectal cancer is a devastating complication affecting 1–30% of patients, with profound clinical, oncological, and psychological implications. Early diagnosis of AL is crucial for timely intervention. We adopted a tiered evidence synthesis approach, prioritizing existing meta-analyses and systematic reviews while supplementing others with relevant primary studies, categorizing biomarkers by healing phase (inflammation, proliferation, and remodeling). Our analysis reveals that C-reactive protein [CRP; cutoff: 140–159 mg/L on postoperative days (POD) 3–5] and procalcitonin (PCT; cutoff: 0.7–1.3 ng/mL on POD3–5) offer high negative predictive value (NPV) in blood, enabling early discharge within enhanced recovery after surgery (ERAS) pathways. Drainage fluid biomarkers [e.g., IL-6, IL-10, matrix metalloproteinase-9 (MMP9), and bile acids (BAs)] demonstrate superior site-specificity, with MMP9 and IL-10 elevation on POD1 showing particular promise for incipient leak detection. Novel approaches include ischemia markers [lactate and intestinal fatty acid binding protein (I-FABP)], collagenolytic microbiota profiling (*Enterococcus faecalis*), and excretion-derived biomarkers [urinary volatile organic compounds (VOCs)], though these require further validation. Critically, drainage fluid analysis capitalizes on existing clinical infrastructure, while combinatorial multi-marker surveillance appears essential to overcome the limitations of singular biomarkers. Future efforts must prioritize integrated diagnostic platforms embedding dynamic biomarker profiling, mechanistic drivers [reactive oxygen species/formyl peptide receptor (ROS/FPR) pathways, enteric dysbiosis, and genetic polymorphisms], and real-time interventions to transition from reactive rescue to personalized AL prevention.

## Introduction

Anastomotic leakage (AL) is a dreaded and serious complication after low anterior resection (LAR) for patients with rectal cancers. The reported incidence of AL in this setting ranges from 1 to 30% worldwide, with a generally accepted AL rate of about 11%^[1–4]^. It has a substantial interruption of the recovery process and following adjuvant therapy plans for these patients, which lead to poor surgical outcomes and increased medical costs^[5,6]^. It is also reported that patients who suffer an AL after LAR have a poor oncological outcome such as local recurrence^[7,8]^. On the other hand, AL also brings the surgeon to heavily psychological stress from colleagues and the patient with their family which make the surgeon the “second victim”[9].

**HIGHLIGHTS**

- CRP and PCT on POD3–5 exhibit high NPV in blood, facilitating early safe discharge within ERAS protocols.
- Drainage fluid biomarkers, particularly IL-10 and MMP9 elevations on POD1, demonstrate superior site-specificity and significant promise for detecting incipient ALs.
- Drainage fluid analysis leverages existing clinical infrastructure, with markers such as BAs, bilirubin, and amylase offering direct evidence of anastomotic compromise via enteric content leakage.
- Novel biomarker strategies encompass ischemia indicators (lactate, I-FABP), collagenolytic microbiota profiling (e.g., *E. faecalis*), and excretion-derived biomarkers (urinary VOCs and fecal calprotectin), though requiring further clinical validation.
- Combinatorial multi-marker surveillance integrating dynamic changes is essential to overcome the limited accuracy inherent in singular biomarker detection for ALs.
- Future efforts must prioritize integrated diagnostic platforms embedding dynamic biomarker profiling, mechanistic drivers (ROS/FPR pathways, dysbiosis, polymorphisms), and real-time interventions to enable personalized leak prevention.

Early diagnosis of ALs after LARs is essential for clinical decision-making, as infections following ALs develop quickly and hamper the rehabilitation of patients, even leading to a high rate of mortality[10]. Thus, early interventions to block the development of septic shock, or even avoid fecal contamination at an earlier stage, will benefit the prognosis of the patients[11].

The symptomatic AL is usually diagnosed several days [although debated, but generally accepted as in postoperative days (POD) 3–6] after LARs^[12,13]^. As initial signs and indications may be varied and unspecific^[14,15]^, it is meaningful to verify the postoperative biomarkers that can have an early alert in continuously monitored patients after LARs.

In this review, we will fully evaluate the available postoperative biomarkers in the early diagnosis of ALs after LARs with a specific focus on their diagnostic utility in identifying incipient leaks during the critical postoperative surveillance period.

## Methods

The current study was compliant with the TITAN Guidelines 2025, and no AI was used[16]. The study originated as a systematic review evaluating biomarkers for AL prediction following LAR. An initial literature search was conducted in PubMed (up to 17 May 2025) using the MeSH terms “colorectal surgery,” “anastomotic leak,” and “biomarkers,” supplemented by entry terms for each concept. This query identified 44 articles (including original studies and reviews) reporting 27 candidate biomarkers, such as C-reactive protein (CRP), procalcitonin (PCT), white cell count (WCC), cytokines, pH, and so on. However, concerns emerged regarding the methodological feasibility of conducting individual systematic reviews for each biomarker. Specifically, generating independent meta-analyses for all 27 biomarkers would require screening thousands of studies, a task impractical due to logistical constraints.

To optimize efficiency, a tiered evidence synthesis approach was adopted. First, we prioritized the latest meta-analyses to consolidate evidence for biomarkers with robust pre-existing syntheses. For biomarkers lacking eligible meta-analyses, systematic searches were conducted using the same database.

The decision against performing full systematic reviews for all biomarkers was multifactorial. First, overlapping efforts with existing meta-analyses were deemed redundant. Second, novel biomarkers lacked sufficient evidence volume to allow meaningful synthesis. Third, resource constraints necessitated prioritizing biomarkers with more immediate clinical relevance.

## Results

The mechanisms of epidermal wound healing have been extensively elucidated and involve three consecutive and overlapping phases: inflammation, proliferation, and remodeling. Theoretically, this process also applies to intestinal anastomotic healing. However, due to the limited ability to visualize the anastomotic site directly, the underlying mechanisms remain incompletely understood[17]. The detailed molecular and cellular events are beyond the scope of this article and are discussed in previous reviews^[18–20]^. In brief, disruption of the delicate balance among inflammation, proliferation, and remodeling may contribute to the development of ALs (Table 1).

**Table 1:** Postoperative biomarkers in the early diagnosis of AL after LAR

Potential biomarker	Sample source	Assay standardization	Analytical method	Turnaround time	Practicality	Characteristics	Data sources
Inflammation-phase markers							
PCT	Blood	Commercially standardized kits protocol	CLIA	TAT ≤ 2 h	Strong	POD3–5 cut-off: 0.7–1.3 ng/ml^[21–23]^	Meta-analysis
CRP	Blood	Commercially standardized kits protocol	Immunoturbidimetry	TAT ≤ 2 h	Strong	POD3–5 cut-off: 140–159 mg/l^[21,22,24]^	Meta-analysis
WCC	Blood	Commercially standardized kits protocol	Impedance methodOptical scattering methodFluorescence staining method	TAT ≤ 0.5 h	Moderate	POD6–7^[22,25,26],^	Meta-analysis
ALB	Blood	Commercially standardized kits protocol	Bromocresol green method	TAT ≤ 2 h	Weak	POD0, 1, 3, 7[27]	Single study
CAR	Blood	Commercially standardized kits protocol	Impedance methodOptical scattering methodFluorescence staining method	TAT ≤ 0.5 h	Moderate	Values ≥ 0.0139–0.049^[28–30]^	Studies
ΔALB	Blood	Commercially standardized kits protocol	Bromocresol green method	TAT ≤ 2 h	Moderate	Values ≥ 27.2% (in female patients with ALs)[31]	Single study
NLR	Blood	Commercially standardized kits protocol	Impedance methodOptical scattering methodFluorescence staining method	TAT ≤ 0.5 h	Moderate	POD3–5 (thresholds: 5.5–8.6), AUC ≥ 0.7; NPV: <7.4 on POD1^[32–43]^	Studies
CLP	Blood	Available but inter-lab variability exists	ELISA	TAT ≤ 6 h	Weak	N/A	Studies
ECC	Blood	Commercially standardized kits protocol	Impedance methodOptical scattering methodFluorescence staining method	TAT ≤ 0.5 h	Weak	POD5[44]	Single study
AMDA	Blood	Research-only methods	LC-MS/MS	TAT ≤ 2 h	Weak	ADMA Δ24/8, cut-off: 0.853 µM, SS = 80%, SP = 82.7%[45]	Single study
IL-6	Drainage fluid	Commercially standardized kits protocol	CLIA	TAT ≤ 1 h	Weak	POD1–3^[46]^	Meta-analysis
IL-1β	Drainage fluid	Available but inter-lab variability exists	ELISA	TAT ≤ 6 h	Weak	N/A	Meta-analysis
TNF-α	Drainage fluid	Available but inter-lab variability exists	ELISA	TAT ≤ 6 h	Weak	POD4[46]	Meta-analysis
IL-10	Drainage fluid	Available but inter-lab variability exists	ELISA	TAT ≤ 6 h	Strong	POD1[47]	Meta-analysis
CRP	Drainage fluid	Commercially standardized kits protocol	Immunturbidimetry	TAT ≤ 2 h	Strong	POD5–7 (thresholds: 70–150 mg/l)[48]	Meta-analysis
LPS	Drainage fluid	Available but inter-lab variability exists	ELISA	TAT ≤ 6 h	Moderate	POD3, higher LPS (5270–6750 pg/ml vs. 1–1848 pg/ml)[49]	Single study
VOCs	Excretion-derived	Research-only methods	Field asymmetric ion mobility spectrometry	TAT ≤ 1 h(DEEPSEEK)	Moderate	POD3,SS = 86%, SP = 93%[50]	Single study
Fecal CLP	Excretion-derived	Available but inter-lab variability exists	ELISA	TAT ≤ 6 h	Weak	POD5[51]	Single study
Proliferation-phase markers							
Lactate	Blood	Commercially standardized kits protocol	Enzymatic catalysis method	TAT ≤ 1 h	Weak	6–8 h after the operation[52]	Single study
I-FABP	Blood	Available but inter-lab variability exists	ELISA	TAT ≤ 6 h	Weak	24 h after the onset of surgery	Studies
IMA	Drainagefluid	Research-only methods	Copper binding assay, Microplate reader Infinite 200 PRO	TAT ≤ 1 h	Moderate	ΔIMA cut-off: 270 FU/g, SS = 100%, SP = 80%[53]	Single study
Lactate	Drainage fluid	Commercially standardized kits protocol	Enzymatic catalysis method	TAT ≤ 1 h	Weak	POD1–5, cut-off: 9.8 mmol/l, SS = 25%, SP = 88%[54]	Meta-analysis
PH	Drainage fluid	Research-only methods	Method 1: Tonometry measured intraluminal CO_2_ to derive intracellular pH via the Henderson–Hasselbalch equation (40–60 min). Method 2: pH meter measured pH at 25℃ (15–20 min)	TAT ≤ 1 h	Moderate	POD3, cut-off: 6.978, SS = 98.7%, SP = 94.7%[55]	Meta-analysis
I-FABP	Excretion-derived	Available but inter-lab variability exists	ELISA	TAT ≤ 6 h	Weak	POD3, cut-off 1.589 ng/ml, SS = 80%, SP = 74%[56]	Single study
Remodeling-phase markers							
MMP9	Drainage fluid	Research-only methods	Immunoassays	TAT ≤ 6 h	Strong	POD1	Meta-analysis
MMP8	Drainage fluid	Research-only methods	MMP-8 was determined by particle-based flow cytometry using F-MAP kits	TAT ≤ 6 h	Weak	N/A	Meta-analysis
Microbial analyses	Drainage fluid	Research-only methods	Quantitative cultures, semi-quantitative real-time PCR, bacterial proliferation assays	TAT ≥ 24 h	Weak	N/A	Meta-analysis
Barrier disruption-associated markers							
Amylase	Drainage fluid	Commercially standardized kits protocol	Enzyme-coupled method	TAT ≤ 2 h	Weak	N/A	Studies
BA	Drainage fluid	Commercially standardized kits protocol	Enzymatic cycling assay	TAT ≤ 1 h	Strong	POD3–5^[57,58]^	Studies
Bilirubin	Drainage fluid	Commercially standardized kits protocol	Enzymatic cycling assay	TAT ≤ 1 h	Strong	POD1 or 3[57]	Single study

ADMA, asymmetric dimethylarginine; AL, anastomotic leakage; ALB, albumin; BA, bile acid; CAR, CRP to ALB ratio; CLIA, chemiluminescence immunoassay; CLP, calprotectin; CRP, C-reactive protein; ECC, eosinophil cell count; F-MAP, fluorokine multi analyte profiling; I-FABP, intestinal fatty acid binding protein; IMA, ischemia-modified ALB; LAR, low anterior resection; LPS, lipopolysaccharides; MMP8, matrix metalloproteinase-8; MMP9, matrix metalloproteinase-9; N/A, not available; NLR, neutrophil-to-lymphocyte ratio; PCR, polymerase chain reaction; PCT, procalcitonin; VOCs, volatile organic compounds; WCC, white cell count.

### Inflammation-phase markers

Inflammation is a central mechanism underlying various pathophysiological processes, including anastomotic healing. Although the specific inflammatory mediators involved in the healing process remain unclear, monitoring postoperative inflammatory levels after LAR is considered potentially useful for the early detection of ALs. Biomarkers such as PCT, CRP, cytokines, complete blood count, and their derived indicators (e.g., neutrophil-to-lymphocyte ratio, NLR) have been extensively investigated for their potential role in predicting ALs.

#### Blood biomarkers

##### Traditional biomarkers

In colorectal surgery, CRP, PCT, and WCC are the most commonly used inflammatory markers for predicting ALs.

Since 2008, studies have shown that CRP might have a significant predictive value for ALs[59]. Based on current meta-analyses, CRP elevation predicts ALs with high discriminatory power, particularly on POD3–5 (thresholds: 140–159 mg/L). Crucially, CRP values below these cut-offs on POD3–5 can exclude ALs, enabling early safe discharge within (enhanced recovery after surgery) pathways, while elevated CRP warrants vigilant assessment or intervention^[21,22,24]^.

Similarly, PCT also has a good ability to distinguish ALs after colorectal surgery and is also considered to have the best predictive utility on POD3–5. Meta-analyses found that the cutoff values on POD3–5 range from 0.7 to 1.3 ng/ml, indicating its reliability in excluding ALs^[21–23]^.

The role of WCC for early diagnosis of ALs was also investigated. In recent meta-analyses, WCC demonstrates limited standalone diagnostic utility for early AL detection due to its delayed elevation (typically on POD6–7) and poor sensitivity/specificity^[22,25,26]^. Its clinical value is primarily observed in combination with other biomarkers, where it may contribute marginally to composite diagnostic models but remains inferior to CRP or PCT for early prediction.

Circulating cytokine also reflects the inflammatory levels after LAR. In a meta-analysis published in 2016, the authors found that most studies focused on peritoneal rather than systemic inflammatory cytokines. A relatively small number of studies did not show the effectiveness of cytokines such as TNF, IL-1β, IL-6, and IL-10[60]. Recently, another meta-analysis targeting IL-10 also indicated that circulating IL-10 could not reliably identify ALs at early stages[61].

Only one study has investigated the role of postoperative albumin (ALB) levels in predicting ALs after colorectal surgery. The authors suggested that reduced serum ALB levels during the early postoperative period may serve as a potentially valuable predictor of ALs in colorectal cancer patients. Nevertheless, the study did not define the threshold of “lower serum albumin levels”[27].

##### Derived biomarkers

Calculated hematologic indices such as the CRP to ALB ratio (CAR), delta albumin (△ALB), and NLR were also studied as potential biomarkers for predicting ALs.

While limited evidence has examined the role of postoperative CAR in early AL detection after colorectal surgery, existing studies uniformly proposed its predictive value. In brief, CAR values ≥0.0139–0.049 emerge as independent predictors for ALs^[28–30]^.

△ALB, defined as (preoperative ALB − nadir ALB within POD2)/preoperative ALB × 100%, indicates postoperative morbidity risk when exceeding specific thresholds^[62,63]^. Our prior study established ∆ALB ≥27.2% as an independent risk factor for ALs in female patients[31].

Postoperative NLR demonstrates significant value as a predictive biomarker for ALs after colorectal surgery, with diagnostic efficacy increasing substantially during POD3–5. The optimal NLR thresholds vary notably between studies (ranging from 5.5 to 8.6) and appear contextual, influenced by surgical approach (e.g., laparoscopic vs. open) and AL grading, though most studies report area-under-curve (AUC) values exceeding 0.70 during peak predictive windows. Notably, both negative predictive value (NPV) at earlier time points (e.g., NLR <7.4 on POD1 excluding ALs with 99.3% NPV) and progressive NLR elevation on POD4–5 serve complementary risk-stratification roles, with POD5 NLR >6.54 showing particular promise for symptomatic AL prediction in rectal cancer cohorts^[32–43]^. However, currently, there is a paucity of meta-analyses that effectively synthesize these data to generate conclusions with a higher level of evidential certainty.

##### Novel biomarkers

Calprotectin (CLP) is a well-studied (both systemic and fecal) inflammatory biomarker[64]. Scattered evidence suggests that monitoring blood CLP after colorectal surgery can help in the early diagnosis of ALs^[65,66]^. However, some studies present opposing views[67].

A recent prospective single-center study suggests the potential utility of eosinophil cell count (ECC) for early AL detection on POD5, but this finding currently lacks sufficient validation to strengthen the existing evidence base[44].

Asymmetric dimethylarginine (ADMA) is associated with ALs and has the potential to predict postoperative complications in CRC patients. Nevertheless, ADMA testing is primarily conducted using liquid chromatography-tandem mass spectrometry (LC-MS/MS) in laboratories, which is costly[45].

#### Drainage fluid biomarkers

As circulating blood biomarkers primarily reflect systemic inflammation rather than the local microenvironment at the anastomotic site (the origin of most ALs) and are susceptible to interference from unrelated systemic inflammatory processes, some studies have shifted focus toward analyzing biomarkers in drainage fluid.

The evaluation of peritoneal cytokines as early warning factors for AL events after LAR has become a hot topic in recent years. A recent meta-analysis investigated IL-1β, IL-6, IL-10, and TNF-α. The results showed that the levels of IL-6 on POD1–3 were significantly higher in patients with AL, while TNF-α only slightly increased on POD4[46]. In another meta-analysis, the authors focused solely on the predictive role of Peritoneal cytokines at POD1. The results showed that the pooled data from four clinical studies indicated that IL-10 was the only peritoneal cytokine that had a predictive effect on AL events on POD1[47].

The latest meta-study findings show that peritoneal CRP shows good diagnostic accuracy for ALs after colorectal surgery, with better performance on POD5–7 and higher thresholds (70–150 mg/l) which means a computed tomography scan is required[48].

An early study investigated the predictive potential of lipopolysaccharides (LPS) in drainage fluid for ALs. The results demonstrated that patients with AL exhibited significantly higher LPS concentrations in the drainage fluid compared to those without AL (5270–6750 vs. 1–1848 pg/ml). Additionally, the total daily excretion of LPS was markedly increased in this group (270–675 vs. 0–92 ng/day)[49]. These findings underscore the specific diagnostic relevance of LPS parameters in the ALs after LAR.

#### Excretion-derived biomarkers

Urine volatile organic compounds (VOCs) show potential as predictive biomarkers for ALs and are involved in the remodeling process of AL[50]. VOCs, produced by colonic fermentation and pathophysiological processes, reflecting alterations in the gut microbiota composition and intestinal inflammation, which are associated with AL.

Fecal CLP also has the potential for early warning. A study involving 11 patients found that patients with AL experienced a significant increase in fecal CLP on POD5. However, the fact that there was only one case of AL in the control group limits the understanding of the results[51].

### Proliferation-phase markers

Adequate blood supply is considered one of the fundamental prerequisites for anastomotic healing. Hypoperfusion and hypoxia in the neorectum may not only disrupt the inflammatory phase but also directly impair cellular proliferation due to insufficient oxygen and nutrient delivery^[68,69]^. Studies have emphasized the importance of early detection of ischemic conditions postoperatively to prevent and manage ALs effectively.

#### Blood biomarkers

Lactate serves as the signature anaerobic metabolite accumulating during impaired tissue perfusion and oxygen deprivation. Few studies indicate that blood lactate levels can serve as an early warning sign for ALs. A single-center study found some promising results regarding serum lactate levels as an early postoperative marker of ALs[52]. They found that as early as 6–8 h after the operation, the lactate levels of patients with AL were significantly higher than those without AL. Interestingly, this did not serve as a warning again in the following days, even though the ALs occurred mainly on POD6–9[52].

Known as a marker for ischemia and a predictor of AL, plasma levels of intestinal fatty acid binding protein (I-FABP) were significantly elevated in patients with AL compared with those without AL at 24 h after the onset of surgery [median 1724 pg/ml (interquartile range (IQR) 1430) vs. 836 pg/ml (IQR 799)][70]. However, some authors reported that I-FABP lacked any distinct potential to detect postoperative bowel complications including ALs[53]. Another study reported that the preoperative I-FABP levels in plasma were higher in patients with AL than those without AL and remained elevated over time postoperatively, but only significantly on POD3[66]. The conflicting results observed across various animal experiments also suggest that the efficacy of this biomarker remains inconclusive, necessitating further studies for rigorous validation^[71,72]^.

Ischemia-modified ALB (IMA) reflects local tissue hypoperfusion at the anastomosis. The difference calculation neutralizes inter-patient variability, unmasking its predictive power. Due to its short half-life, IMA concentrations showed no significant deviation from preoperative to postoperative periods. However, △IMA (postoperative IMA within POD0 − preoperative IMA) with a cut-off value of 270 fluorescent units per gram emerged as a significant AL predictor, indicating that IMA dynamics could offer a novel “rule-out” strategy for AL. Although the confirmation ability is limited, it is a valuable addition to the AL diagnostic toolkit[53].

#### Drainage fluid biomarkers

Elevated lactate in drainage fluid may serve as a real-time biomarker of tissue hypoperfusion, with its dynamic changes correlating with the severity of regional oxygen debt. A systematic review showed that increased intraperitoneal lactate concentration within the POD1–5 was significantly associated with clinical ALs but with low predictive values[54].

Low pH reflects local tissue ischemia (reduced perfusion) and bacterial metabolism (organic acid production), both driving AL pathogenesis. A systematic review found that in studies focusing on colorectal patients, pH monitoring could be a real-time, cost-effective AL screening tool, particularly valuable for early intervention in high-risk colorectal surgeries[55].

#### Excretion-derived biomarkers

As a biomarker of intestinal epithelial injury, elevated I-FABP offers non-invasive detection potential for ALs. A multicenter study showed that on postoperative day three, urinary I-FABP levels in patients with AL were significantly higher, with an AUROC of 0.775, a sensitivity of 80%, and a specificity of 74% at the optimal cutoff of 1.589 ng/ml[56].

### Remodeling-phase markers

The deposition and degradation of collagens undeniably play pivotal roles in anastomotic healing. Excessive collagenolytic activity may precipitate AL, as evidenced by Shogan *et al*’s identification of AL-associated *Enterococcus faecalis* strains (high collagenase producers) that exacerbate collagen breakdown through two synergistic mechanisms: first, direct collagen I degradation via bacterial gelatinase; and second, conversion of host pro-MMP9 into its active form via GelE metalloproteinase and SprE serine protease-dependent proteolytic cleavage, thereby amplifying pathological matrix degradation[73].

A meta-analysis by Edomskis *et al*, encompassing three studies measuring MMP9 in postoperative drainage fluid, demonstrated that elevated MMP9 levels on POD1 exhibited early diagnostic potential for AL. However, analysis of two subsequent studies focusing on POD3 MMP9 measurements failed to yield significant predictive utility[74]. Notably, a separate meta-analysis by Reeves *et al*, incorporating overlapping datasets, concluded that total MMP9 levels lacked efficacy for early AL detection[47]. These conflicting findings likely stem from limited sample sizes and heterogeneity in postoperative monitoring time points, which obscure the definitive interpretations of the results.

Exploratory investigations evaluating other MMPs (e.g., MMP-1, -2, -3, -7, -8, -13) and tissue inhibitors of metalloproteinases (TIMPs) have been sparse, with only isolated reports suggesting potential biomarker candidates[75].

Dysregulated MMP activation may disrupt the dynamic equilibrium between collagen deposition and degradation at anastomotic sites, prompting efforts to profile collagenase-producing bacterial species in drainage fluid. Recently, a systematic review identified four studies utilizing drainage fluid microbial analyses (quantitative cultures, semi-quantitative real-time polymerase chain reaction (PCR), bacterial proliferation assays) to detect *Escherichia coli, Klebsiella, Pseudomonas* spp., *E. faecalis*, and *Bacteroides* as AL-associated collagenolytic pathogens^[76–80]^. This systematic review consolidates clinical evidence that collagenase-producing species are enriched in patients with AL, though methodological heterogeneity across studies precludes definitive conclusions on their causal role or strain-specific contributions to anastomotic failure. Critical knowledge gaps persist regarding: (i) standardization of microbial profiling methodologies, (ii) threshold bacterial loads diagnostic of AL, and (iii) temporal limitations of culture-based detection for timely clinical intervention.

### Barrier disruption-associated markers

The disruption of the intestinal anastomotic healing process may lead to impairment of anastomotic integrity, allowing digestive enzymes or metabolites typically confined within the intestinal lumen to extravasate through defective intestinal walls and subsequently appear in postoperative drainage fluid. Therefore, the presence of these biomarkers in drainage fluid constitutes direct evidence of enteric content leakage and serves as laboratory indicators for anastomotic compromise. Potentially diagnostic biomarkers include amylase, lipase, bilirubin, pancreatic elastase, urobilinogen, and bile acids (BAs). Nevertheless, only sporadic studies have evaluated their utility in the early diagnosis of ALs, with limited evidence supporting their clinical validity.

### Digestive enzymes

In a series of studies conducted by Clark *et al*, they reported a significant increase in amylase levels in peritoneal or transanal drainage fluid in patients who finally developed ALs after colorectal surgery^[81,82]^. Recently, Ishiyama *et al* reported similar results in a two-center prospective study[83].

### Metabolites

Paasch *et al* evaluated the effectiveness of bilirubin, urobilin, pancreatic elastase, and BA in drainage as predictor markers of AL after CRC surgery. The results showed that the concentration of BA in patients with ALs differed from those without ALs on POD4, while the concentration of bilirubin on POD1 or 3 was different in patients with or without ALs[57]. Gao *et al* evaluated the drainage fluid BA in 419 patients undergoing laparoscopic LAR and found that the BA in the AL group was significantly higher than that in the non-AL group on POD3 and 5[58].

Although the analysis of drainage fluid collected via transanal or transabdominal tube has been considered a promising strategy for early AL detection due to its high specificity, standardized threshold criteria remain unvalidated. This limitation hinders definitive diagnosis in clinically ambiguous cases and requires further validation of its diagnostic utility. Furthermore, the presence of diverting stomas may confound interpretation, rendering this approach potentially impractical for patients undergoing prophylactic stoma.

## Discussion

Our study systematically evaluated postoperative biomarkers with potential utility in the early diagnosis of ALs after LAR. The identified biomarkers were categorized into three distinct groups: blood biomarkers, drainage fluid biomarkers, and excretion-derived biomarkers, all of which play roles across multiple phases of healing (Table 1). Consequently, vigilant postoperative surveillance of these markers holds promise for early identification of ALs.

This review substantially advances the literature by implementing a tiered evidence synthesis methodology to systematically address the complexity of diverse biomarkers for AL detection after LAR. Unlike conventional reviews, our approach prioritized meta-analyses for established biomarkers while conducting targeted systematic searches for emerging candidates, thus optimizing resource allocation without sacrificing comprehensiveness. Critically, we establish a novel dual-axis framework that classifies biomarkers both by biological healing phases (inflammation, proliferation, and remodeling) and sampling sources (blood, drainage fluid, and excreta), revealing previously overlooked synergies across phases relevant to AL pathogenesis. By addressing contemporary controversies, such as the diagnostic utility of drainage-derived markers versus the efficacy of prophylactic drains, and by introducing TDTs as anatomically optimized conduits for biomarker sampling, we have effectively bridged biomarker science with surgical practice. Collectively, these methodologically rigorous and clinically translational advances provide a roadmap for transitioning from reactive AL management to mechanism-targeted prevention.

Blood biomarker-based monitoring serves as a widely adopted strategy for early detection of ALs. Both CRP and PCT have demonstrated robust diagnostic utility, exhibiting high efficacy in both ruling out and ruling in ALs. In contrast, WCC functions primarily as an adjunctive marker requiring combinatorial analysis with other indicators, while circulating cytokines show limited diagnostic value. Concurrently, substantial research has focused on the NLR, which displays significant predictive potential yet requires further validation through more well-designed studies. Several promising blood biomarkers, including the CAR, perioperative serum ALB decline (ΔALB), CLP, ECC, ADMA, △IMA, lactate, and I-FABP, await more conclusive clinical verification.

In contrast, studies suggest that drainage fluid biomarkers hold greater promise for future clinical application, as they directly reflect the peri-anastomotic microenvironment. Moreover, specimen collection is less burdensome with existing drains (reducing patient discomfort compared to frequent blood draws). Multiple markers in drainage fluid, including CRP, IL-6, IL-10, TNF-α, gut microbial profiling, LPS, lactate, pH, amylase, BA, bilirubin, and MMP9, demonstrate early-warning potential for detecting ALs in the postoperative period. Notably, both MMP9 and IL-10 exhibit predictive value as early as POD1, highlighting their status as the most promising candidates.

Building upon the inherent advantage of convenient drainage fluid sampling, researchers have adopted enhanced longitudinal tracking protocols. Recently, Jessernig and colleagues designed a macromolecular network sensing element to continuously detect the presence of digestive enzymes in drainage from patients undergoing abdominal surgery[84]. They reported a 100% success rate in AL diagnosis using the sensing element in a validation set of 32 samples[84]. Intriguingly, emerging evidence challenges the prophylactic efficacy of pelvic drains in anastomotic protection, promoting judicious reduction of their postoperative utilization[85]. However, should the early-warning capacity of drainage fluid biomarkers for ALs be validated, surgeons may revisit drain deployment strategies.

Emerging excretion-derived biomarkers, including urinary VOCs, I-FABP, and fecal CLP, have demonstrated diagnostic potential for anastomotic monitoring. However, VOC profiling necessitates specialized field asymmetric ion mobility spectrometry platforms, entailing both dedicated instrumentation and complex analytical workflows that currently confine application to research settings. Compounding these limitations, fecal output remains notoriously sparse during the initial postoperative days post-LAR. Collectively, these constraints highlight the imperative for methodological refinement of biomarker assays, mandating robust validation through rigorously designed clinical studies. On the other hand, robust clinical data now substantiate the risk-reduction effect of transanal drainage tubes (TDTs) following LAR^[86,87]^, rendering TDT-derived fluid/exudate analysis a promising avenue for AL surveillance – given their superior anatomic proximity to the anastomotic microenvironment compared to pelvic drains. Longitudinal monitoring of VOCs in TDT-derived flatus/fecal emissions may pioneer a novel surveillance paradigm for rapid AL detection^[88,89]^.

Although the detection of a single biomarker has a certain predictive value, its accuracy is still insufficient to meet clinical needs. Future studies should consider the combined detection of multiple biomarkers, and the dynamic changes monitoring, so as to develop comprehensive predictive models to improve the accuracy and reliability of diagnosis^[44,90–92]^.

Building upon mechanistic insights into intestinal anastomotic healing processes, future investigations should capitalize on emerging molecular targets to develop novel biomarkers for early AL detection. Emerging evidence implicates specific reactive oxygen species (ROS) derivatives and formyl peptide receptor (FPR) activation as critical regulators of wound healing cascades, positioning these mediators as putative contributors to colorectal AL pathogenesis[93]. On the other hand, leveraging the characteristics of the gut microbiota and genetic polymorphisms to enhance the accuracy of AL predictions is also a promising direction^[94,95]^.

Finally, we note that despite growing studies on monitoring biomarkers, current investigations remain predominantly focused on validation studies. From a mechanistic perspective, successful clinical translation necessitates platforms capable of early detection to initiate preemptive interventions. Such systems can regulate pathological cascades and restore homeostatic balance during intestinal anastomotic healing, ultimately enabling mechanism-targeted personalized prevention of ALs. This represents a critical trajectory for future investigation.

## Conclusion

The evolving landscape of biomarker studies demonstrates significant potential for enhancing early AL detection following LAR, yet critical challenges persist. Collectively, blood-based markers like CRP/PCT offer practical diagnostic utility, while drainage fluid biomarkers deliver superior specificity by directly interrogating the anastomotic microenvironment. Novel approaches encompass ischemia markers, microbial profiling, intraluminal biomarkers, and excretion-derived biomarkers; however, these methods necessitate further validation to establish their clinical utility. Building upon mechanistic insights into AL pathogenesis, including signaling pathway analyses (e.g., ROS and FPRs), microbial compositional shifts, enteric dysbiosis, and genetic polymorphism considerations, future research must prioritize the accelerated development of integrated diagnostic platforms. These systems should facilitate real-time, mechanism-targeted interventions to attenuate pathological cascades and restore anastomotic homeostasis, thereby transitioning AL management from reactive rescue strategies to personalized prevention approaches.

## Data Availability

No datasets were generated or analyzed during the current study. Data sharing is not applicable to this article.

## Research registration unique identifying number (UIN)

This review article does not involve any human subjects or clinical trials. No registration is required.

## References

[1] Hoek VT Buettner S Sparreboom CL. A preoperative prediction model for anastomotic leakage after rectal cancer resection based on 13.175 patients. Eur J Surg Oncol 2022;4848:2495–501. 10.1016/j.ejso.2022.06.016 35768313

[2] Snijders HS Wouters MW van Leersum NJ. Meta-analysis of the risk for anastomotic leakage, the postoperative mortality caused by leakage in relation to the overall postoperative mortality. Eur J Surg Oncol 2012;38:1013–19. 22954525 10.1016/j.ejso.2012.07.111

[3] Paun BC Cassie S MacLean AR Dixon E Buie WD. Postoperative complications following surgery for rectal cancer. Ann Surg 2010;251:807–18. 20395841 10.1097/SLA.0b013e3181dae4ed

[4] Zhao S Zhang L Gao F. Transanal drainage tube use for preventing anastomotic leakage after laparoscopic low anterior resection in patients with rectal cancer: a randomized clinical trial. JAMA Surg 2021;156:1151–58. 34613330 10.1001/jamasurg.2021.4568 PMC8495603

[5] Chiarello MM Fransvea P Cariati M Adams NJ Bianchi V Brisinda G. Anastomotic leakage in colorectal cancer surgery. Surg Oncol 2022;40:101708. 35092916 10.1016/j.suronc.2022.101708

[6] Tsai YY Chen WT. Management of anastomotic leakage after rectal surgery: a review article. J Gastrointest Oncol 2019;10:1229–37. 31949944 10.21037/jgo.2019.07.07 PMC6955017

[7] Koedam TWA Bootsma BT Deijen CL. Oncological outcomes after anastomotic leakage after surgery for colon or rectal cancer: increased risk of local recurrence. Ann Surg 2022;275:e420–e427. 32224742 10.1097/SLA.0000000000003889

[8] Arron MNN Greijdanus NG Bastiaans S. Long-term oncological outcomes after colorectal anastomotic leakage: a retrospective dutch population-based study. Ann Surg 2022;276:882–89. 35930021 10.1097/SLA.0000000000005647 PMC9534056

[9] Baigrie RJ Stupart D. Coping with anastomotic leaks: harder as one gets older? Br J Surg 2023. doi:10.1093/bjs/znac455. PMC10364549 36637232

[10] Bostrom P Haapamaki MM Rutegard J Matthiessen P Rutegard M. Population-based cohort study of the impact on postoperative mortality of anastomotic leakage after anterior resection for rectal cancer. BJS Open 2019;3:106–11. 30734021 10.1002/bjs5.50106 PMC6354192

[11] Rhodes A Evans LE Alhazzani W. Surviving sepsis campaign: international guidelines for management of sepsis and septic shock: 2016. Intensive Care Med 2017;43:304–77. 28101605 10.1007/s00134-017-4683-6

[12] Nasirkhan MU Abir F Longo W Kozol R. Anastomotic disruption after large bowel resection. World J Gastroenterol 2006;12:2497–504. 16688793 10.3748/wjg.v12.i16.2497 PMC4087980

[13] van Helsdingen CP Jongen AC de Jonge WJ Bouvy ND Derikx JP. Consensus on the definition of colorectal anastomotic leakage: a modified Delphi study. World J Gastroenterol 2020;26:3293–303. 32684743 10.3748/wjg.v26.i23.3293 PMC7336323

[14] Sutton CD Marshall LJ Williams N Berry DP Thomas WM Kelly MJ. Colo-rectal anastomotic leakage often masquerades as a cardiac complication. Colorectal Dis 2004;6:21–22. 14692947 10.1111/j.1463-1318.2004.00574.x

[15] Erb L Hyman NH Osler T. Abnormal vital signs are common after bowel resection and do not predict anastomotic leak. J Am Coll Surg 2014;218:1195–99. 24680576 10.1016/j.jamcollsurg.2013.12.059

[16] Agha R Mathew G Rashid R. Transparency In The reporting of Artificial INtelligence – the TITAN guideline. Premier J Sci 2025;10:100082.

[17] Lam A Fleischer B Alverdy J. The biology of anastomotic healing-the unknown overwhelms the known. J Gastrointest Surg 2020;24:2160–66. 32524361 10.1007/s11605-020-04680-w PMC7446770

[18] Foppa C Ng SC Montorsi M Spinelli A. Anastomotic leak in colorectal cancer patients: new insights and perspectives. Eur J Surg Oncol 2020;46:943–54. 32139117 10.1016/j.ejso.2020.02.027

[19] Witte MB Barbul A. Repair of full-thickness bowel injury. Crit Care Med 2003;31:S538–46. 12907884 10.1097/01.CCM.0000081436.09826.A4

[20] Rosendorf J Klicova M Herrmann I. Intestinal anastomotic healing: what do we know about processes behind anastomotic complications. Front Surg 2022;9:904810. 35747439 10.3389/fsurg.2022.904810 PMC9209641

[21] Bona D Danelli P Sozzi A. C-reactive protein and procalcitonin levels to predict anastomotic leak after colorectal surgery: systematic review and meta-analysis. J Gastrointest Surg 2023;27:166–79. 36175720 10.1007/s11605-022-05473-z

[22] Dost W Rasully MQ Zaman MN. Predictive biomarkers for the early detection of anastomotic leaks in colorectal surgeries: a systematic review. Cureus 2024;16:e74616. 39735081 10.7759/cureus.74616 PMC11679563

[23] Xu Z Zong R Zhang Y Chen J Liu W. Diagnostic accuracy of procalcitonin on POD3 for the early diagnosis of anastomotic leakage after colorectal surgery: a meta-analysis and systematic review. Int J Surg 2022;100:106592. 35257965 10.1016/j.ijsu.2022.106592

[24] Yeung DE Peterknecht E Hajibandeh S Hajibandeh S Torrance AW. C-reactive protein can predict anastomotic leak in colorectal surgery: a systematic review and meta-analysis. Int J Colorectal Dis 2021;36:1147–62. 33555423 10.1007/s00384-021-03854-5

[25] Selvamani TY Shoukrie SI Malla J. Predictors that identify complications such as anastomotic leak in colorectal surgery: a systematic review. Cureus 2022;14:e28894. 36105895 10.7759/cureus.28894 PMC9451042

[26] Almutairi FM. Role of biomarkers in the diagnosis of anastomotic leakage after colorectal surgery: a systematic review and meta-analysis. Cureus 2024;16:e62432. 39011204 10.7759/cureus.62432 PMC11249052

[27] Shimura T Toiyama Y Hiro J. Monitoring perioperative serum albumin can identify anastomotic leakage in colorectal cancer patients with curative intent. Asian J Surg 2018;41:30–38. 27451010 10.1016/j.asjsur.2016.07.009

[28] Sugimoto A Toyokawa T Miki Y. Preoperative C-reactive protein to albumin ratio predicts anastomotic leakage after esophagectomy for thoracic esophageal cancer: a single-center retrospective cohort study. BMC Surg 2021;21:348. 34548054 10.1186/s12893-021-01344-7 PMC8454123

[29] Paliogiannis P Deidda S Maslyankov S. C reactive protein to albumin ratio (CAR) as predictor of anastomotic leakage in colorectal surgery. Surg Oncol 2021;38:101621. 34126521 10.1016/j.suronc.2021.101621

[30] Harada T Numata M Izukawa S. C-reactive protein-to-albumin ratio as a risk factor for anastomotic leakage after anterior resection for rectal cancer with intraoperative use of indocyanine green fluorescence imaging. Surg Endosc 2024;38:4236–44. 38858251 10.1007/s00464-024-10940-6

[31] Zheng H Li F Xie X Zhao S Huang B Tong W. Preservation versus nonpreservation of the left colic artery in anterior resection for rectal cancer: a propensity score-matched analysis. BMC Surg 2022;22:164. 35538516 10.1186/s12893-022-01614-y PMC9092824

[32] Paliogiannis P Deidda S Maslyankov S. Blood cell count indexes as predictors of anastomotic leakage in elective colorectal surgery: a multicenter study on 1432 patients. World J Surg Oncol 2020;18:89. 32375770 10.1186/s12957-020-01856-1 PMC7204308

[33] Pantoja Pachajoa DA Gielis M Palacios Huatuco RM. Neutrophil-to-lymphocyte ratio vs C-reactive protein as early predictors of anastomotic leakage after colorectal surgery: a retrospective cohort study. Ann Med Surg Lond 2021;64:102201. 33763228 10.1016/j.amsu.2021.102201 PMC7973302

[34] Liu YJ Gao CQ Wang GC Wang YC Lu XZ Han GS. [The clinical values of neutrophil-to-lymphocyte ratio as an early predictor of anastomotic leak in postoperative rectal cancer patients]. Zhonghua Zhong Liu Za Zhi 2020;42:70–73. 32023773 10.3760/cma.j.issn.0253-3766.2020.01.011

[35] Walker PA Kunjuraman B Bartolo DCC. Neutrophil-to-lymphocyte ratio predicts anastomotic dehiscence. ANZ J Surg 2018. doi:10.1111/ans.14369. 29377500

[36] Josse JM Cleghorn MC Ramji KM. The neutrophil-to-lymphocyte ratio predicts major perioperative complications in patients undergoing colorectal surgery. Colorectal Dis 2016;18:O236–42. 27154050 10.1111/codi.13373

[37] Mik M Dziki L Berut M Trzcinski R Dziki A. Neutrophil to lymphocyte ratio and c-reactive protein as two predictive tools of anastomotic leak in colorectal cancer open surgery. Dig Surg 2018;35:77–84. 28132052 10.1159/000456081

[38] Song G Guo H Jiang C Shi Y Liu M. Magnetic nanoparticles detection of C-reactive protein combined with neutrophil to lymphocyte ratio to predict the occurrence of anastomotic fistula after rectal cancer surgery. Cell Mol Biol (Noisy-le-grand) 2022;68:116–22. 36800824 10.14715/cmb/2022.68.8.21

[39] Zhang F Qiao S Yao N. Anastomotic rings and inflammation values as biomarkers for leakage of stapled circular colorectal anastomoses. Diagnostics (Basel) 2022;12. doi:10.3390/diagnostics12122902. PMC9777459 36552909

[40] Tan F Xu K Qi X. Neutrophil-to-lymphocyte ratio as an early predictor of symptomatic anastomotic leakage in patients after rectal cancer surgery: a propensity score-matched analysis. J Pers Med 2022;13:93. 36675754 10.3390/jpm13010093 PMC9862085

[41] Agnello L Buscemi S Di Buono G. Drainage fluid LDH and neutrophil to lymphocyte ratio as biomarkers for early detecting anastomotic leakage in patients undergoing colorectal surgery. Clin Chem Lab Med 2024;62:967–78. 37988156 10.1515/cclm-2023-1164

[42] Gordiichuk M Myasoyedov S. Laboratory predictors for diagnosing colorectal anastomotic leakage. Exp Oncol 2024;46:146–53. 39396169 10.15407/exp-oncology.2024.02.146

[43] Ioannidis A Tzikos G Smprini A. Negative and positive predictors of anastomotic leakage in colorectal cancer patients-the case of neutrophil-to-lymphocyte ratio. Diagnostics (Basel) 2024;14:1806. 39202294 10.3390/diagnostics14161806 PMC11353382

[44] Rama NJG Lages MCC Guarino MPS. Usefulness of serum C-reactive protein and calprotectin for the early detection of colorectal anastomotic leakage: a prospective observational study. World J Gastroenterol 2022;28:2758–74. 35979163 10.3748/wjg.v28.i24.2758 PMC9260864

[45] Bednarz-Misa I Fleszar MG Zawadzki M. L-Arginine/NO pathway metabolites in colorectal cancer: relevance as disease biomarkers and predictors of adverse clinical outcomes following surgery. J Clin Med 2020;9:1782. 32521714 10.3390/jcm9061782 PMC7355854

[46] Qi XY Liu MX Xu K. Peritoneal cytokines as early biomarkers of colorectal anastomotic leakage following surgery for colorectal cancer: a meta-analysis. Front Oncol 2021;11:791462. 35127496 10.3389/fonc.2021.791462 PMC8815457

[47] Reeves N Vogel I Ghoroghi A Ansell J Cornish J Torkington J. Peritoneal cytokines as a predictor of colorectal anastomotic leaks on postoperative day 1: a systematic review and meta-analysis. Tech Coloproctol 2022;26:117–25. 34817744 10.1007/s10151-021-02548-y

[48] Vun T Wu Z Chea C Liu W Tao R Deng Y. C-reactive protein in peritoneal fluid for predicting anastomotic leakage after colorectal cancer surgery: a systematic review and meta-analysis. J Clin Med 2025;14:2099. 40142907 10.3390/jcm14062099 PMC11942750

[49] Junger W Junger WG Miller K. Early detection of anastomotic leaks after colorectal surgery by measuring endotoxin in the drainage fluid. Hepatogastroenterology 1996;43:1523–29. 8975959

[50] Plat VD Bootsma BT Neal M. Urinary volatile organic compound markers and colorectal anastomotic leakage. Colorectal Dis 2019;21:1249–58. 31207011 10.1111/codi.14732

[51] Hiraki M Tanaka T Sato H. The analysis of fecal calprotectin as a diagnostic marker for anastomotic leakage after rectal cancer surgery: a pilot study. J Surg Case Rep 2023;2023:rjad432. 37525751 10.1093/jscr/rjad432 PMC10387369

[52] Janez J Horvat G Jerin A Grosek J. The significance of blood and peritoneal fluid biochemical markers in identifying early anastomotic leak following colorectal resection-findings from a single-center study. Medicina (Kaunas) 2022;58. doi:10.3390/medicina58091253. PMC9502513 36143930

[53] Hyspler R Ticha A Kaska M. Markers of perioperative bowel complications in colorectal surgery patients. Dis Markers 2015;2015:428535. 26788017 10.1155/2015/428535 PMC4693001

[54] Ellebaek MB Daams F Jansson K. Peritoneal microdialysis as a tool for detecting anastomotic leakage in patients after left-side colon and rectal resection. A systematic review. Scand J Gastroenterol 2018;53:1625–32. 30457391 10.1080/00365521.2018.1533033

[55] Walshaw J Hugh K Helliwell J Burke J Jayne D. Perianastomotic pH monitoring for early detection of anastomotic leaks in gastrointestinal surgery: a systematic review of the literature. Surg Innov 2025;32:180–95. 39773077 10.1177/15533506241313168 PMC11894859

[56] Plat VD Derikx JPM Jongen AC. Diagnostic accuracy of urinary intestinal fatty acid binding protein in detecting colorectal anastomotic leakage. Tech Coloproctol 2020;24:449–54. 32107682 10.1007/s10151-020-02163-3

[57] Paasch C Rink S Steinbach M. Bilirubin, urobilinogen, pancreas elastase and bile acid in drain fluid. The GBUP-study: analysis of biomarkers for a colorectal anastomotic leakage. Ann Med Surg Lond 2018;35:44–50. 30294427 10.1016/j.amsu.2018.09.008 PMC6170325

[58] Gao L Zhang T Chen X. Bile acid in drainage fluid for early diagnosis of anastomotic leakage and safe discharge after minimally invasive rectal cancer resection: a prospective cohort study. Surgery 2025;178:108949. 39657328 10.1016/j.surg.2024.10.032

[59] Matthiessen P Henriksson M Hallbook O Grunditz E Noren B Arbman G. Increase of serum C-reactive protein is an early indicator of subsequent symptomatic anastomotic leakage after anterior resection. Colorectal Dis 2008;10:75–80. 17666099 10.1111/j.1463-1318.2007.01300.x

[60] Sparreboom CL Wu Z Dereci A. Cytokines as early markers of colorectal anastomotic leakage: a systematic review and meta-analysis. Gastroenterol Res Pract 2016;2016:3786418. 27051416 10.1155/2016/3786418 PMC4804081

[61] Villegas-Coronado L Villegas-Coronado K Villegas Coronado D. Peritoneal and systemic interleukin-10 as early biomarkers for colorectal anastomotic leakage following surgery in colorectal cancer patients: a systematic review and meta-analysis. Pol Przegl Chir 2023;96:135–42. 38348991 10.5604/01.3001.0053.9836

[62] Ge X Dai X Ding C. Early postoperative decrease of serum albumin predicts surgical outcome in patients undergoing colorectal resection. Dis Colon Rectum 2017;60:326–34. 28177996 10.1097/DCR.0000000000000750

[63] Issangya CE Msuya D Chilonga K. Perioperative serum albumin as a predictor of adverse outcomes in abdominal surgery: prospective cohort hospital based study in Northern Tanzania. BMC Surg 2020;20:155. 32664910 10.1186/s12893-020-00820-w PMC7362485

[64] Jukic A Bakiri L Wagner EF Tilg H Adolph TE. Calprotectin: from biomarker to biological function. Gut 2021;70:1978–88. 34145045 10.1136/gutjnl-2021-324855 PMC8458070

[65] Cikot M Kones O Gedikbasi A. The marker C-reactive protein is helpful in monitoring the integrity of anastomosis: plasma calprotectin. Am J Surg 2016;212:53–61. 26606896 10.1016/j.amjsurg.2015.06.018

[66] Reisinger KW Poeze M Hulsewe KW. Accurate prediction of anastomotic leakage after colorectal surgery using plasma markers for intestinal damage and inflammation. J Am Coll Surg 2014;219:744–51. 25241234 10.1016/j.jamcollsurg.2014.06.011

[67] van den Bos J Jongen A Melenhorst J. Near-infrared fluorescence image-guidance in anastomotic colorectal cancer surgery and its relation to serum markers of anastomotic leakage: a clinical pilot study. Surg Endosc 2019;33:3766–74. 30710314 10.1007/s00464-019-06673-6 PMC6795629

[68] Han G Ceilley R. Chronic wound healing: a review of current management and treatments. Adv Ther 2017;34:599–610. 28108895 10.1007/s12325-017-0478-y PMC5350204

[69] Han X Ju LS Irudayaraj J. Oxygenated wound dressings for hypoxia mitigation and enhanced wound healing. Mol Pharm 2023;20:3338–55. 37338289 10.1021/acs.molpharmaceut.3c00352 PMC10324602

[70] Peters EG Dekkers M van Leeuwen-hilbers FW. Relation between postoperative ileus and anastomotic leakage after colorectal resection: a post hoc analysis of a prospective randomized controlled trial. Colorectal Dis 2017;19:667–74. 27943617 10.1111/codi.13582

[71] Popescu GA Jung I Cordos BA Azamfirei L Hutanu A Gurzu S. Intestinal fatty acid-binding protein, as a marker of anastomotic leakage after colonic resection in rats. Rom J Morphol Embryol 2018;59:1075–81. 30845287

[72] Bosmans JW Jongen AC Birchenough GM. Functional mucous layer and healing of proximal colonic anastomoses in an experimental model. Br J Surg 2017;104:619–30. 28195642 10.1002/bjs.10456

[73] Shogan BD Belogortseva N Luong PM. Collagen degradation and MMP9 activation by *Enterococcus faecalis* contribute to intestinal anastomotic leak. Sci Transl Med 2015;7:286ra68. 10.1126/scitranslmed.3010658 PMC5027898 25947163

[74] Edomskis P Goudberg MR Sparreboom CL. Matrix metalloproteinase-9 in relation to patients with complications after colorectal surgery: a systematic review. Int J Colorectal Dis 2021;36:1–10. 32865714 10.1007/s00384-020-03724-6 PMC7782374

[75] Pasternak B Matthiessen P Jansson K Andersson M Aspenberg P. Elevated intraperitoneal matrix metalloproteinases-8 and −9 in patients who develop anastomotic leakage after rectal cancer surgery: a pilot study. Colorectal Dis 2010;12:e93–8. 19508511 10.1111/j.1463-1318.2009.01908.x

[76] Jorgensen AB Jonsson I Friis-Hansen L Brandstrup B. Collagenase-producing bacteria are common in anastomotic leakage after colorectal surgery: a systematic review. Int J Colorectal Dis 2023;38:275. 38038731 10.1007/s00384-023-04562-y PMC10692267

[77] Fouda E El Nakeeb A Magdy A Hammad EA Othman G Farid M. Early detection of anastomotic leakage after elective low anterior resection. J Gastrointest Surg 2011;15:137–44. 20978948 10.1007/s11605-010-1364-y

[78] Sparreboom CL Komen N Rizopoulos D. A multicentre cohort study of serum and peritoneal biomarkers to predict anastomotic leakage after rectal cancer resection. Colorectal Dis 2020;22:36–45. 31344302 10.1111/codi.14789 PMC6973162

[79] Bilgin IA Hatipoglu E Aghayeva A. Predicting value of serum procalcitonin, C-reactive protein, drain fluid culture, drain fluid interleukin-6, and tumor necrosis factor-alpha levels in anastomotic leakage after rectal resection. Surg Infect (Larchmt) 2017;18:350–56. 28394749 10.1089/sur.2016.222

[80] Komen N Slieker J Willemsen P. Polymerase chain reaction for *Enterococcus faecalis* in drain fluid: the first screening test for symptomatic colorectal anastomotic leakage. The appeal-study: analysis of parameters predictive for evident anastomotic leakage. Int J Colorectal Dis 2014;29:15–21. 24122105 10.1007/s00384-013-1776-8

[81] Clark DA Edmundson A Steffens D Harris C Stevenson A Solomon M. Drain fluid amylase as a biomarker for the detection of anastomotic leakage after rectal resection without a diverting ileostomy. ANZ J Surg 2022;92:813–18. 34994080 10.1111/ans.17461

[82] Clark DA Edmundson A Steffens D Radford-Smith G Solomon M. Multicenter study of drain fluid amylase as a biomarker for the detection of anastomotic leakage after ileal pouch surgery without a diverting ileostomy. Dis Colon Rectum 2022;65:1335–41. 35358101 10.1097/DCR.0000000000002376

[83] Ishiyama Y Hirano Y Yamato M. Drainage fluid amylase as a biomarker for the detection of anastomotic leakage after low anterior resection of rectal cancer: a two-center study. Cancer Diagn Progn 2024;4:802–07. 39502605 10.21873/cdp.10399 PMC11534043

[84] Jessernig A Anthis AHC Vonna E. Early detection and monitoring of anastomotic leaks via naked eye-readable, non-electronic macromolecular network sensors. Adv Sci (Weinh) 2024;11:e2400673. 38775058 10.1002/advs.202400673 PMC11304232

[85] Irani JL Hedrick TL Miller TE. Clinical practice guidelines for enhanced recovery after colon and rectal surgery from the American society of colon and rectal surgeons and the society of American gastrointestinal and endoscopic surgeons. Dis Colon Rectum 2023;66:15–40. 36515513 10.1097/DCR.0000000000002650 PMC9746347

[86] Tamura K Uchino M Nomura S. Updated evidence of the effectiveness and safety of transanal drainage tube for the prevention of anastomotic leakage after rectal low anterior resection: a systematic review and meta-analysis. Tech Coloproctol 2024;28:71. 38916755 10.1007/s10151-024-02942-2

[87] Zhao S Hu K Tian Y Xu Y Tong W. Role of transanal drainage tubes in preventing anastomotic leakage after low anterior resection: a meta-analysis of randomized controlled trials. Tech Coloproctol 2022;26:931–39. 35915290 10.1007/s10151-022-02665-2

[88] Arasaradnam RP Covington JA Harmston C Nwokolo CU. Review article: next generation diagnostic modalities in gastroenterology – gas phase volatile compound biomarker detection. Aliment Pharmacol Ther 2014;39:780–89. 24612215 10.1111/apt.12657

[89] Moura PC Raposo M Vassilenko V. Breath volatile organic compounds (VOCs) as biomarkers for the diagnosis of pathological conditions: a review. Biomed J 2023;46:100623. 37336362 10.1016/j.bj.2023.100623 PMC10339195

[90] Martin G Dupre A Mulliez A Prunel F Slim K Pezet D. Validation of a score for the early diagnosis of anastomotic leakage following elective colorectal surgery. J Visc Surg 2015;152:5–10. 25577712 10.1016/j.jviscsurg.2014.12.002

[91] den Dulk M Witvliet MJ Kortram K. The DULK (Dutch leakage) and modified DULK score compared: actively seek the leak. Colorectal Dis 2013;15:e528–33. 24199233 10.1111/codi.12379

[92] den Dulk M Noter SL Hendriks ER. Improved diagnosis and treatment of anastomotic leakage after colorectal surgery. Eur J Surg Oncol 2009;35:420–26. 18585889 10.1016/j.ejso.2008.04.009

[93] Bachmann R Leonard D Delzenne N Kartheuser A Cani PD. Novel insight into the role of microbiota in colorectal surgery. Gut 2017;66:738–49. 28153961 10.1136/gutjnl-2016-312569

[94] Hajjar R Gonzalez E Fragoso G. Gut microbiota influence anastomotic healing in colorectal cancer surgery through modulation of mucosal proinflammatory cytokines. Gut 2023;72:1143–54. 36585238 10.1136/gutjnl-2022-328389

[95] Wu M Mi B Liu L. Genetic polymorphisms, biomarkers and signaling pathways associated with septic shock: from diagnosis to therapeutic targets. Burns Trauma 2024;12:tkae006. 38716051 10.1093/burnst/tkae006 PMC11074594

